# Evaluation of phage assay for rapid phenotypic detection of rifampicin resistance in *Mycobacterium tuberculosis*

**DOI:** 10.1186/1476-0711-5-11

**Published:** 2006-04-21

**Authors:** Sergio Luis Yzquierdo, Dihadenys Lemus, Miguel Echemendia, Ernesto Montoro, Ruth McNerney, Anandi Martin, Juan Carlos Palomino

**Affiliations:** 1 Instituto de Medicina Tropical "Pedro Kouri", La Habana, Cuba; 2 London School of Hygiene & Tropical Medicine, London, UK; 3 Institute of Tropical Medicine, Antwerp, Belgium

## Abstract

**Background:**

Conventional methods for susceptibility testing require several months before results can be reported. However, rapid methods to determine drug susceptibility have been developed recently. Phage assay have been reported as a rapid useful tools for antimicrobial susceptibility testing. The aim of this study was to apply the Phage assay for rapid detection of resistance on *Mycobacterium tuberculosis *strains in Cuba.

**Methods:**

Phage D29 assay was performed on 102 *M. tuberculosis *strains to detect rifampicin resistance. The results were compared with the proportion method (gold standard) to evaluate the sensitivity and specificity of Phage assay.

**Results:**

Phage assay results were available in 2 days whereas Proportion Methods results were obtain in 42 days. A total of 44 strains were detected as rifampicin resistant by both methods. However, one strains deemed resistant by Proportion Methods was susceptible by Phage assay. The sensitivity and specificity of Phage assay were 97.8 % and 100% respectively.

**Conclusion:**

Phage assay provides rapid and reliable results for susceptibility testing; it's easy to perform, requires no specialized equipment and is applicable to drug susceptibility testing in low income countries where tuberculosis is a major public health problem.

## Background

Tuberculosis (TB) remains a major cause of morbidity and mortality worldwide. It is estimated that about one-third of the world's population is infected with *Mycobacterium tuberculosis*, more than eight million new cases of active TB occur annually and the estimated global annual mortality from this disease is close to two million people [1]. Multidrug-resistant tuberculosis (MDR-TB), caused by strains resistant to at least isoniazid (INH) and rifampicin (RMP), is considered an emergent disease as well as the consequence of inadequate treatment [2]. WHO has estimated that approximately 460.000 MDR-TB cases occur each year [3]. Estimated prevalence of MDR-TB ranges from 0% in some western countries to 14.2% in Kazakhstan and Israel, high prevalence has also been observed in the Russian Federation (Tomsk Oblast, 13.7%); Uzbekistan (Karakalpakstan, 13.2%), Estonia (12.2%), China (Liaoning Province, 10.4% and Henan Province, 7.8%), Lithuania (9.4%), Latvia (9.3%), and Ecuador (6.6%) [4].

Currently available techniques for susceptibility testing are culture based, and include the proportion method (PM), resistance ratio and absolute concentration method. Conventional methods require several months before results can be reported. Delays in reporting results lead to prolong an inadequate treatment and may sustain transmission of drug resistant disease. More rapid detection may be obtained by using dyes to indicate bacterial growth such as MTT and Resazurin and a number of assays are currently being evaluated [5]. These methods show high sensitivity and specificity values to detect MDR-TB at lower cost than more conventional approaches [1].

Rapid molecular methods have been developed recently, some of which are commercially available [5]. Based on PCR followed by reverse hybridization to identify either specific mutations or wild-type sequences the INNO-LiPA Rif TB assay (Innogenetics, Ghent, Belgium) detects resistance to rifampicin [6], while the GenoType^® ^MTBDR test (Hain Lifescience Gmbh, Germany) detects resistance to both rifampicin and isoniazid within one working day [7]. However, their expense and the requirement for specialist skills and equipment have prevented their adoption in countries with less favorable living conditions, where TB is an important health problem.

Mycobacteriophages (phages) were initially investigated as tools for investigating resistance to anti-tuberculosis drugs over 25 years ago [8,9]. More recently mycobacteriophage-based techniques have been reported for detection of viable bacilli in clinical specimens as well as for antimicrobial susceptibility testing [10,11]. Phages are unable to replicate in the presence of drugs such as RMP that disrupt the mechanism of replication of the host bacteria. However, in drug resistant strains replication can proceed. The resulting phage can be visualized on indicator plates as clear areas (plaques) within a lawn of fast growing mycobacteria [12]. This ingenious method, which does not require specialist laboratory skills or sophisticated equipment, appears to offer robust detection of RMP resistance [13]. Initial reports suggested that resistance to rifampicin could be detected in 4 days [10], but this has since been reduced to less than 48 hours [14]. Furthermore, the assay is available as a commercial diagnostic kit as well as an 'in-house' version [15].

The aim of this study was to apply the Phage assay for rapid detection of RMP resistance on *M. tuberculosis *strains in Cuba.

## Methods

### Strains

A total of 102 *M. tuberculosis *strains from the collection at the Tuberculosis National Reference Laboratory of Institute de Medicina Tropical "Pedro Kourf" were studied in a blinded manner. Two *M. tuberculosis *reference strains were used as control of the assay, *M. tuberculosis *H_37_Rv rifampicin sensitive (ATCC 27294) and *M. tuberculosis *(ATCC 35838) rifampicin resistant. *Mycobacterium smegmatis *mc^2 ^155 was used to produce indicator plates.

### Indicator plates

Indicator plates were produced as described previously [16]. One hundred microliters of M. *smegmatis *mc^2 ^155 which was stored in Middlebrook 7H9 (Difco) at -70°C, was spread on a plate which contained 1.5 % agar (Difco) with Middlebrook 7H9 and 10% oleic acid albumin dextrose catalase (OADC; Difco). The plate was incubated for three days at 37°C and then, one colony was taken and placed in 300 mL of Middlebrook 7H9 with 10% OADC and incubated for 2 days at 37°C. To prepare the plates 10% stationary phase *M. smegmatis *culture (100 mL) was added in a medium which contained 15 g agar (1.5 %) and 800 mL of Middlebrook 7H9 with 100 mL OADC (10 %). Then approximately 25 mL of this mix was poured onto Petri dishes. Once set, the indicator plates were stored for up to 2 weeks at 4°C.

### Phage

Mycobacteriophage D29 was obtained as described previously [16]. One hundred microliters of mycobacteriophage which were stored in Middlebrook 7H9 with 10% OADC and ImM CaCl_2 _at 4°C were spread in an indicator plate and incubated overnight at 37°C. Ten milliliters of Middlebrook 7H9 with 10% OADC and ImM CaCl_2 _were added and incubated overnight at 37°C. Then, the medium was filtered through a sterile 0.45 μm filter and stored in 0.1 mL at 4°C for as long as 6 months. The mycobacteriophage stock was quantified by pipetting 10 μL aliquots of serial dilutions (10 fold) on an indicator plate and the titre of plaque forming units (pfu) calculated.

### Antibiotic

RMP (Sigma) was made up as stock solution at 1 mg/mL in dimethylsulfoxide (BDH, England) and stored at -70°C in 0.5 mL vials until use. Further dilutions were made in Middlebrook 7H9 with 10% OADC and ImM CaCl_2_.

### Phage assay

The assay was carried out as described Wilson et al. [10]. To reduce cost, the assay was performed in 0.5 mL vials. Seventy five microliters of M. tuberculosis (McFarland No 1 turbidity) and 75 μL of RMP (20 ug/mL) were placed in vials and incubated during 24 h at 37°C. Fifty microliters of mycobacteriophage D29 (10^8 ^pfu/mL) were added into the vials and it were incubated for 90 min at 37°C. Then, the extracellular viruses were destroyed with 100 μL of phagicidal agent ferrous ammonium sulfate at 30 mM (Merck) [11] and finally, the mycobacteriophages were detected by addition 10 μL of the samples on the lawn indicator plate after overnight incubation at 37°C. A growth control containing no drug was included for each strain. The strains were considered resistant if lytic plaques were observed on the indicator plate from those samples treated with RMP; strains were considered susceptible if no plaques were observed following drug treatment.

### Proportion method

The PM was carried out on tubes with Löwenstein-Jensen medium according to the standard procedure with the recommended critical concentration of RMP 40 mg/mL [17]. This method was used as the gold standard to evaluate the sensitivity and specificity of Phage assay.

## Results

**Figure 1:**
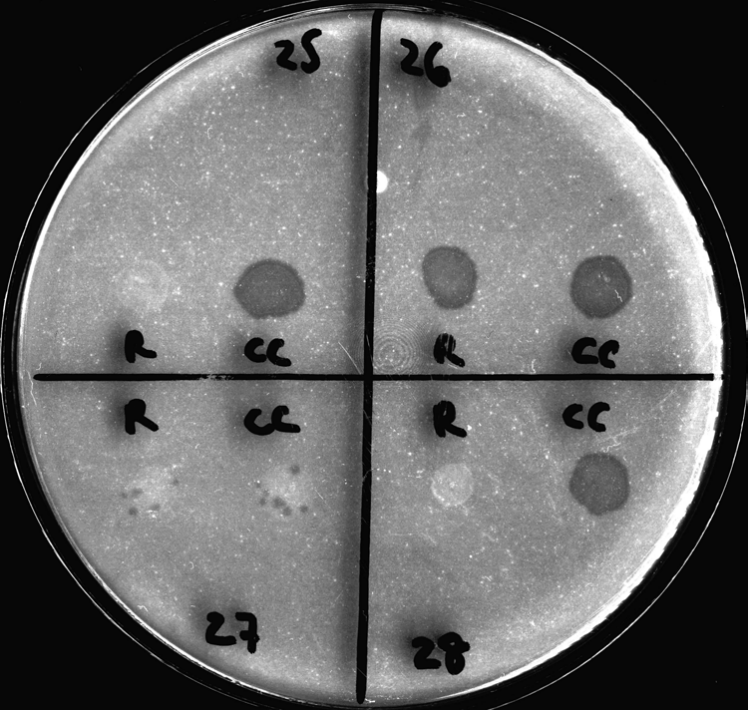
Interpretation of the phage assay in the indicator plates. The resistance strains (26 and 27) showed lysis on the indicator plates in both the RPM treated and control samples, whereas the sensitive strains (25 and 28) had lysis only in the growth (no drug) control.

The Phage assay and PM were performed with the purpose to detect RMP resistance on 102 *M. tuberculosis *strains. Phage assay results were available in 2 days whereas PM results were obtained in 42 days. Table 1 shows the results obtained by both methods. Interpretation of the results was easy and clear. The resistant strains showed lysis on the indicator plates in both the RPM treated and control samples, whereas the sensitive strains had lysis only in the growth (no drug) control (Figure 1).

**Table 1:** Comparison of Phage assay with Proportion Method to detect Rifampicin-resistant

Proportion Method	Phage assay			
	
	Resistant	Susceptible	Sensitivity	Specificity
Resistant	44	1	97.8 %	100%
Susceptible	0	57		

Of 45 strains resistant by the PM, 44 were resistant by the Phage assay. One strain deemed resistant by the PM was susceptible by Phage assay. On the other hand, all the 57 susceptible strains by the PM were also classified as susceptible by Phage assay.

The overall comparison of the Phage assay with the PM produced only one discordant result. The sensitivity of the Phage assay in detecting RMP resistance was 97.8 % whereas the specificity achieved was 100 %.

## Discussion

Rifampicin is the most potent sterilizing drug available and a key component for TB treatment. Since RMP resistance is considered a marker of MDR in high level countries, it would be helpful for low resources countries to have a simple and inexpensive test than can rapidly detect resistance to RMP [5,18].

The diagnosis of MDR-TB is based on in vitro drug susceptibility testing. Conventional culture-based methods are still in routine use in most countries. Susceptibility testing is typically performed on cultures isolated from clinical specimens. These methods requires 3–4 weeks to obtain the results and, combined with the time required for primary isolation, may take several months to obtain the results. Automatic culture systems, such as Bactec 460 have reduced time to several weeks but the machine, the tubes, and the additives are all expensive and generate radioactive waste. Other commercial tests such as the MGIT (mycobacterial growth indicator tube) and molecular tools such as LIPA (line probe assay) have been developed but are expensive and impractical for routine use in resource poor settings [5].

In this study, the Phage assay was performed for rapid detection of RMP resistance in 102 strains of *M. tuberculosis *and the results were compared with the gold standard PM recommended by WHO. Results of the Phage assay were available approximately 40 days earlier than PM that which is significant in terms of patient management [15].

One discrepant result was obtained in which the phage test misidentified a strain as susceptible whereas PM determined it as resistant. When tested by the colorimetric MTT and Resazurin Microtitre Assays [18] this strain showed resistance to RMP >2 mg/L by both methods. The failure of D29 to detect resistant bacilli in this sample may be due to experimental error or could be ascribed to technical problems regarding phage replication which will not proceed in bacilli that are dormant or where replication has been disrupted. It is possible that the presence of aggregates of bacteria may have deterred phage access to the rifampicin resistant bacilli in this sample. To date, no strains of *M. tuberculosis *or *M. smegmatis *have been identified that lack the binding site for D29 on their cell wall [13]. When reporting results some authors have applied proportional analysis to the numbers of plaque forming units observed. However, most researchers, including us, prefer criteria in which the presence of a simple plaque is indicative of resistance [14,19,20].

The results of the present research are similar to those obtained in Argentina and Brazil by Simboli *et al*. and da Silva *et al*. [19,21]. Our result showed a good agreement with PM and it confirms the excellent alternative for rapid drug susceptibility testing in poor countries. On the other hand, the assay not only can be applied to RMP but also to INH, ethambutol, pyrazinamide, streptomycin, and ciprofloxacin [22,23]. For more rapid screening of patients it has recently been demonstrated that phages can detect RMP resistance directly from smear positive sputum samples [24]. However, further studies are required to determine the sensitivity and reliability of this method.

## Conclusion

The Phage assay doesn't require any specialized equipment or reagents, only basic additional consumable items, such as Petri dishes and pipettes are needed. Besides this, the assay is easy to perform and does not require training in specialist skills. Furthermore, the results of our study indicate the potentiality of the simple and inexpensive Phage assay for control programs in countries with high levels of MDR-TB. The assay could be use, with minor modifications, as a rapid screen for antimicrobial resistance to the principal drugs against TB.

## Abbreviations

TB: Tuberculosis

MDR-TB: Multidrug-resistant tuberculosis

INH: Isoniazid

RMP: Rifampicin

WHO: World Health Organization

PM: Proportion Method

PCR: Polymerase Chain Reaction

OADC: oleic acid albumin dextrose catalase

PFU: plaque performing unit

## Authors' contributions

SLY carried out the Phage assay, participated in the development of the protocol and had substantial contributions in the analysis and interpretation of data. Also, he was involved in the writing of the manuscript. DL carried out the PM and Phage assay, participated in the development of the protocol. Also, she had substantial contributions in the analysis and interpretation of data and she was involved in the revision of the content of the document. ME carried out the PM and supplied the strains from the collection at the Laboratory. EM supervised the design and execution of the study and contributed to the writing and revision of the manuscript. Moreover, he had substantial contributions in the analysis and interpretation of data and gave the final approval of the version to be published. RM, AM and JCP participated in the development of the protocol, they were involved in the revision of the content of document and helped to the writing of the manuscript and they had substantial contributions in the analysis and interpretation of data. All authors read and approved the final manuscript.
